# The role of GATA2 in adult hematopoiesis and cell fate determination

**DOI:** 10.3389/fcell.2023.1250827

**Published:** 2023-11-14

**Authors:** Iris J. A. Peters, Emma de Pater, Wei Zhang

**Affiliations:** Department of Hematology, Erasmus MC Cancer Institute, Rotterdam, Netherlands

**Keywords:** GATA2, hematopoietic stem cell (HSC), GATA2 deficiency syndrome, myelodysplastic syndrome (MDS), acute myeloic leukemia (AML), immune deficiency

## Abstract

The correct maintenance and differentiation of hematopoietic stem cells (HSC) in bone marrow is vital for the maintenance and operation of the human blood system. GATA2 plays a critical role in the maintenance of HSCs and the specification of HSCs into the different hematopoietic lineages, highlighted by the various defects observed in patients with heterozygous mutations in GATA2, resulting in cytopenias, bone marrow failure and increased chance of myeloid malignancy, termed GATA2 deficiency syndrome. Despite this, the mechanisms underlying GATA2 deficiency syndrome remain to be elucidated. The detailed description of how GATA2 regulates HSC maintenance and blood lineage determination is crucial to unravel the pathogenesis of GATA2 deficiency syndrome. In this review, we summarize current advances in elucidating the role of GATA2 in hematopoietic cell fate determination and discuss the challenges of modeling GATA2 deficiency syndrome.

## 1 Introduction

**FIGURE 1:**
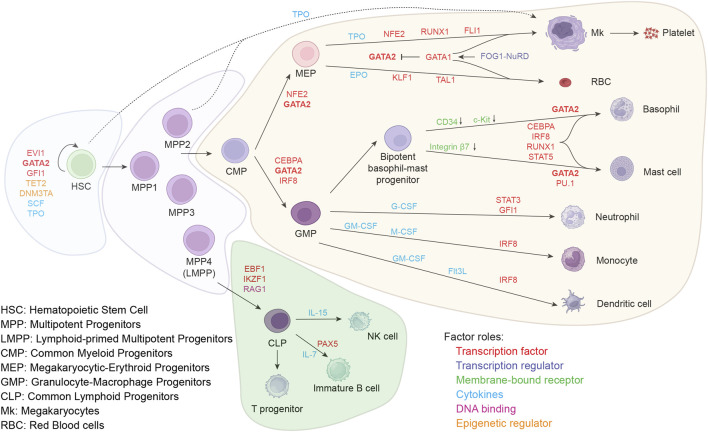
Lineage differentiation in BM and important regulators of the process.

The adult hematopoietic system is derived from hematopoietic stem cells (HSCs) situated within the bone marrow (BM). According to Waddington’s epigenetic theory, various blood cell types originate from unstable stem/progenitor cells and eventually fall into a stable cell fate development track (Waddington, 1957; Ladewig et al., 2013) producing myeloid and lymphoid cells for immunity, erythrocytes for oxygen and carbon dioxide transport and platelets for coagulation. The process of hematopoietic lineage formation resembles a branching tree structure (Figure 1). Within the human bone marrow, the apex point of this classical branching structure is self-renewing HSCs which are typically characterized by the phenotype CD49f^+^CD90^+^CD45RA^–^CD34^+^CD38^–^LIN^–^ (Notta et al., 2011).

Numerous genetic mutations result in hematopoietic disorders with unbalanced lineage output, such as RUNX1 mutations, leading to familial platelet disorder (Preudhomme et al., 2009), IRF8 mutations resulting in mononuclear phagocytes-related human primary immunodeficiencies (Hambleton et al., 2011), and mutations in ELANE or HAX1 resulting in severe congenital neutropenia (Ye et al., 2011). A prime example is GATA2 deficiency syndrome (Hahn et al., 2011; Hsu et al., 2011; Ostergaard et al., 2011; Spinner et al., 2014; Calvo and Hickstein, 2023). GATA2 deficiency syndrome, caused by germline mutations in the hematopoietic transcription factor GATA2, stands out because multiple lineages can be affected and patients often present with monocytopenia, B cell deficiency, NK (natural killer) cell deficiency and Dendritic Cell deficiency (Dickinson et al., 2011; Novakova et al., 2016). Neutropenia also occurs in GATA2 deficiency patients (Pasquet et al., 2013) and inversions of the CD4/CD8 T cell ratio have been reported (Mutsaers et al., 2013; Ganapathi et al., 2015), indicating that GATA2 plays a crucial role as a key component within the BM hematopoietic hierarchy, orchestrating the differentiation and maintenance of diverse hematopoietic cell lineages. Furthermore, GATA2 deficiency syndrome patients have a high predisposition to develop (pediatric) myelodysplastic syndrome (MDS) or acute myeloid leukemia (AML) with a median age of onset of 17 years (Wlodarski et al., 2016; Homan et al., 2021), however, before the onset of malignancy, the disease is also life-threatening due to anemia, bleeding disorders, or immunodeficiency with nontuberculous mycobacterial infections (NTM), fungal infections, and human papillomavirus (HPV) infections (Spinner et al., 2014; Ganapathi et al., 2015; Calvo and Hickstein, 2023). Therefore, it is vital to understand the role of GATA2 in the molecular determinants of hematopoietic cell fate.

A schematic representation of the classical tree-like hematopoiesis model shows formation of the various lineages in human bone marrow. The HSC population forms the apex of this hierarchical model, and differentiates into distinct lineages. Important modulators of the lineage choices are depicted, such as transcription factors, transcription regulators, membrane-bound receptors, cytokines, and epigenetic regulators.

## 2 The role of GATA2 in HSC self-renewal and differentiation

To preserve the hematopoietic system, HSCs are required to self-renew. To preserve the self-renewal capacity of HSCs in the BM microenvironment, a variety of extracellular and intracellular factors must provide support. Extrinsically, different cellular factors, such as stem cell factor (SCF) and thrombopoietin (TPO), organize a coordinated extracellular microenvironment to preserve the self-renewal and maintenance of HSCs (Stoffel et al., 1999; Ema et al., 2000; Fox et al., 2002; Yoshihara et al., 2007; Mendelson and Frenette, 2014; Kokkaliaris et al., 2016). Intrinsically, the self-renewal of HSCs is influenced by multiple transcription factors, including GATA2, GFI1, and EVI1, and epigenetic regulatory molecules, such as TET2 and DNM3TA (Zhu and Emerson, 2002; Hock et al., 2004; Huck et al., 2014; Jeong et al., 2018; Xavier-Ferrucio and Krause, 2018; Aljoufi et al., 2022). GATA2 has various roles in supporting the maintenance of adult HSC characteristics. Complete knockout of *Gata2* in mice results in apoptosis of HSCs (Tsai et al., 1994; de Pater et al., 2013; Gao et al., 2013). In proliferating HSCs, *Gata2* expression is activated by EVI1 and it was shown that haploinsufficiency of Gata2 impairs cell cycle in mice (Ling et al., 2004; Yuasa et al., 2005). Interestingly, a Gata2 reporter mouse model showed that all HSCs have intermediate levels of Gata2 and that Gata2 is variable in multipotent hematopoietic progenitor cells, suggesting that different levels of Gata2 influence lineage determination (Kaimakis et al., 2016). Interestingly, Gata2 protein levels were observed to be constantly fluctuating in embryonic definitive HSPC formation during the endothelial-to-hematopoietic transition (EHT), indicating that Gata2 expression is a dynamic process in HSPC generation, likely required for normal lineage differentiation. Gata2 heterozygous animals displayed reduced Gata2 protein fluctuations and this may be the underlying cause of the lineage differentiation defects (Eich et al., 2018). Together, this shows that the gene dosage of Gata2 in embryonic and adult HSPCs is crucial for normal lineage differentiation.

As HSCs differentiate into various hematopoietic lineages, they receive extrinsic and intrinsic signals that prompt specialization towards specific blood cell lineages, resulting in the gradual reduction of self-renewal and multi-potency. Extrinsically, cytokines, including Flt3L, SCF, granulocyte colony-stimulating factor (G-CSF), interleukin-1 (IL-1), interleukin-3 (IL-3), interleukin-6 (IL-6), and interleukin-11 (IL-11), coordinate the development of multipotent progenitors (MPPs) from HSCs. *SCF* expression can be detected in several niche cells, including osteoblasts, endothelial cells and LepR^+^ perivascular stromal cells, suggesting the importance of the microenvironment for HSC maintenance and differentiation (Ding et al., 2012; Zhou et al., 2014; Zhou et al., 2017).

MPPs are heterogeneous with distinct transcriptomic characteristics. Combined single-cell barcoding and transcriptional analysis reported that MPPs in mice could be further defined as MPP1, MPP2, MPP3, and MPP4, which showed different features and lineage bias through cell fate decisions (Rodriguez-Fraticelli et al., 2018). The first lineage priming separates myeloid and lymphoid differentiation from erythroid lineage differentiation (Notta et al., 2016; Belluschi et al., 2018). MPPs are gradually directed to the myeloid and lymphoid lineages (Velten et al., 2017). Upregulation of *Rag1*, *Ikzf1*, and *Ebf1* in the MPP population will lead to lymphoid bias, while the upregulation of *Cebpa* and *Irf8* will lead to myeloid bias (Wolfler et al., 2010; Pietras et al., 2015; Lenaerts et al., 2022). Although differentiation does not occur in a clear step-wise manner, several progenitors like Lymphoid-Primed Multipotent Progenitors (LMPPs), Common Myeloid Progenitors (CMPs) and Common Lymphoid Progenitors (CLPs) can be recognized and will be discussed as such.

### 2.1 Erythroid differentiation

Megakaryocytes (Mk) and erythrocytes are the first lineage to bifurcate from MPPs driven by the lineage-priming module of GATA2-NFE2 (Sanjuan-Pla et al., 2013; Belluschi et al., 2018) and are generated from megakaryocyte-erythroid progenitors (MEPs). EPO induces the specialization of MEPs to erythroid cells (Li et al., 2014). As development progresses, the size of erythroid cells gradually decreases, the nucleus gradually condenses, and terminally enucleates to form mature red blood cells (Sankaran et al., 2012; Wang S. et al., 2022b; Soboleva and Miharada, 2022). GATA1 plays a vital role in erythropoiesis as it is related to essential erythrocyte functions, including heme synthesis, globin synthesis/switch, and enucleation. As reported, GATA1 interacts with all known erythrocyte development-related genes (Ferreira et al., 2005; Ludwig et al., 2022).

Downregulation of GATA2 is an essential signal for Mk and erythroid lineage commitment. Downregulation of GATA2 results in a chromatin occupancy switch from GATA2 bound loci to GATA1 together with FOG1 bound loci. This change in chromatin occupation, termed “GATA factor switching,” is indispensable for differentiation towards Mk/erythrocytes and blocks mast cell differentiation (Tsai and Orkin, 1997; Grass et al., 2003; Anguita et al., 2004; Dore et al., 2012).

GATA1 is involved in the precise downregulation of GATA2 expression. GATA2 expression is promoted by the direct binding of GATA2 itself to the upstream *WGATAR* motif (Grass et al., 2003). During erythroid development, upregulation of GATA1 leads to the recruitment of FOG1 and NuRD, forming the GATA1-FOG1-NuRD complex that acts to repress GATA2 transcription through *WGATAR* motif occupation. As a result, the GATA2 level is gradually reduced alongside the increase in GATA1 expression during erythropoiesis (Ferreira et al., 2007; Gao et al., 2010; Gregory et al., 2010; Mancini et al., 2012).

For Mk maturation and platelet release, TPO induces the specialization of MEPs to Mks (Ng et al., 2012). During Mk development, the cell size continues to increase, while DNA replicates, but does not undergo mitosis. Eventually, this forms a large and lobulated mature Mk, which then releases platelets into the circulation. For Mk maturation, GATA1 and FOG1 (ZFPM1) can mediate the expression of the Mk marker CD41 (Mancini et al., 2012; Gekas and Graf, 2013). NFE2, FLI1, and RUNX1 are also critical for the terminal maturation of Mks (Zang et al., 2016). Transplantations in mice has further clarified the lineage specification of the erythroid and megakaryocyte lineage, indicating that TPO induces direct Mk development from HSCs, bypassing other hierarchical progenitors (Sanjuan-Pla et al., 2013). Although the downregulation of GATA2 is required for normal Mk differentiation, atypical Mk were observed in BM from germline GATA2 mutated patients (Ganapathi et al., 2015). This could point to a defect in correct downregulation of GATA2 in GATA2 deficiency patients. In a zebrafish model for Gata2 deficiency, such a mechanism was observed, where heterozygous loss of Gata2b (orthologue of GATA2) resulted in dysplastic erythroid lineage cells caused by excess of open chromatin at the Gata2b locus (Gioacchino et al., 2021b).

### 2.2 Myeloid differentiation

Common myeloid progenitors (CMPs) have the capacity to form CFU-GEMM (colony-forming unit-granulocyte erythroid macrophage megakaryocyte) in colony-forming assays under the influence of granulocyte-macrophage colony-stimulating factor (GM-CSF) and G-CSF (Pietras et al., 2015; Regan-Komito et al., 2020).

Throughout the progression from MPPs to CMPs, cytokines such as Flt3L, SCF, and IL-3 continue to sustain the proliferative capacity of progenitor cells. Under the mediation of SCF and interleukin-4 (IL-4), CMPs undergo differentiation into mast cells (Okayama and Kawakami, 2006). The development of basophils is facilitated by the sustained activity of GM-CSF and IL-3. Under the influence of GM-SCF and IL-3, CMPs develop into a mature Basophil. Single cell research showed that the differentiation of basophils and mast cells is closely linked and they share a bipotent basophil-mast cell progenitor (Hamey et al., 2021; Wanet et al., 2021; Miyake et al., 2023). In these progenitors, loss of CD34 and downregulation of c-Kit indicate differentiation in the direction of basophils, while loss of integrin β7 in c-Kit^+^ cells indicate differentiation in the direction of mast cells. The cooperation between GATA2 and PU.1 stimulates the lineage commitment of mast cells (Walsh et al., 2002). Additionally, several transcription factors, including CEBPA, IRF8, GATA2, RUNX1, and STAT5, play a critical role in the maturation of mast cells and basophils (Li et al., 2015; Sasaki et al., 2015). The highest expression of GATA2 in the hematopoietic system is detected in basophils and specifically the GATA2-STAT5 axis is critical for both mast cell and basophil differentiation (Zon et al., 1991; Li et al., 2015).

Monocytes and granulocytes are derived from the same progenitors, GMPs, downstream of CMPs (Rodrigues et al., 2008; Guilliams et al., 2018). The cytokines, SCF, IL-3, GM-CSF, and M-CSF, are all equally important for the differentiation of GMPs (Metcalf, 2008; Ushach and Zlotnik, 2016). M-CSF and GM-CSF induces monocyte/macrophage specialization from GMPs, while G-CSF induces neutrophil lineage specialization from GMPs via STAT3 signaling (Semerad et al., 2002; Irandoust et al., 2007; Ushach and Zlotnik, 2016; Kawano et al., 2017). The generation of dendritic cells from bone marrow progenitors is highly reliant on Flt3/Flt3L and is distinct from the further differentiation of monocytes into dendritic cells (Guilliams et al., 2014; Murphy and Murphy, 2022). Downstream of cytokines, key lineage-restricted transcription factors are critical for the hematopoietic cell fate determination, such as *Irf8* (monocytes/dendritic cells) and *Gfi1* (neutrophils) (Wei et al., 2008; Olsson et al., 2016; Sichien et al., 2016; Murakami et al., 2021). Additionally, heterozygous Gata2 mutated mice displayed GMP defects. It was shown that GATA2 plays a critical regulatory role in GMP function through the GATA2-HES1 signaling axis (Rodrigues et al., 2008).

The critical role that GATA2 plays in GMP formation, myeloid differentiation and maturation easily explains the regulatory mechanisms behind the dendritic cell deficiency, monocytopenia, and neutropenia frequently observed in patients that have been diagnosed with GATA2 deficiency syndromes (Hsu et al., 2011; Spinner et al., 2014; Calvo and Hickstein, 2023). Therefore, it is surprising that these phenotypes are not easily modeled in mice. This could be due to the absence of secondary injuries like infections or the fact that mice are bred in a congenic background. Interestingly, these cytopenic phenotypes have been modeled using zebrafish, where homozygous deletion of Gata2b leads to neutropenia (Gioacchino et al., 2021a; Avagyan et al., 2021), and loss of the intronic enhancer of *Gata2a* results in monocytopenia and neutropenia (Dobrzycki et al., 2020; Mahony et al., 2023), providing direct insights into the molecular effects of GATA2 mutation in blood lineage differentiation in the hematopoietic system. This, however, does not explain why only specific lineages are affected in zebrafish, e.g., the monocyte lineage in Gata2a enhancer mutant zebrafish, while this lineage is unaffected in Gata2b mutant zebrafish. This suggests that GATA2 is not only required for the GMP cell state, but also plays a role in the lineage differentiation choice these cells make.

### 2.3 Lymphoid differentiation

The adaptive immune system is indispensable for protection from invasion of pathogens, by recognition of non-self. The lymphoid lineage is derived from the common lymphoid progenitor (CLP) and this cell gives rise to natural killer (NK) cells, the B cell lineage and T cell lineage. The upregulation of CD122, a receptor for interleukin-15 (IL-15) in NK cell progenitors underscores the pivotal role IL-15 plays in orchestrating essential processes such as proliferation, metabolism, and survival throughout NK cell differentiation (Huntington et al., 2007; Carotta et al., 2011; Anton et al., 2015). Recently, the importance of the GATA2-TGF-b1 axis in regulating NK cell development was reported. In this axis, GATA2 controls the production of TGF-b1 in NK cells, showing the influence of GATA2 on NK formation and explaining the phenotype seem in patients (Wang D. et al., 2022a).

IL-7 acts as the primary cytokine of B cell lineage differentiation in fetal and adult stages in mice, although IL-7 independent B cell differentiation is described in human, highly reliant on FLT3 ligand (Carvalho et al., 2001; Jensen et al., 2008; von Muenchow et al., 2016). Besides cytokines, intracellular factors will also facilitate B lymphopoiesis. The simultaneous expression of *Lhx2*, *Hox9* and *Runx1* could drive B lineage fate commitment using pluripotent stem cells (PSCs) as cell source (Zhang et al., 2022). Bone marrow is the primary development location of immature B cells. Subsequently immature B cells can give rise to secondary B cell development in secondary lymphoid organs, like the spleen and tissue lymph nodes, where immature B cells continuously develop into naïve mature B cells (Mueller and Germain, 2009). In secondary lymphoid organs, naïve mature B cells differentiate into plasma cells (PCs), germinal center (GC) B cells, and GC-independent memory B cells (MBCs) by antigen receptor signaling in combination with T follicular helper cells (Ochiai et al., 2013; Krautler et al., 2017; Ise et al., 2018). PAX5 is a pivotal transcription factor for B lineage decision, but shows downregulation during PC generation (Chan et al., 2017; Calderon et al., 2021). Furthermore, high level of Irf4 is required for PC differentiation in mice (Ochiai et al., 2013). The differentiation of GC B cell can also be initiated by the dynamic expression of Irf4, while GC B cell generated from naïve B would develop into various B cell subpopulations like memory B and long-lived plasma cells undergoing complicated primary and secondary immune response (Ochiai et al., 2013; Akkaya et al., 2020).

Progenitor-T cells are double negative (DN) for CD4 and CD8 and can be divided into several well-defined cell stages orderly following the expression of CD44 and CD25: DN1 with CD44^+^ CD25^-^, DN2 with CD44^+^ CD25^+^, DN3 with CD44^-^CD25^+^, DN4 with CD44^-^ CD25^-^ (Olariu et al., 2021). These progenitor-T cell subpopulations are transcriptionally and functionally distinct. Proliferation mainly occurs in DN1 and DN2, while T cell receptor gene arrangement starts from DN3 (Olariu et al., 2021). A single-cell study in mice indicated that the “early T cell precursor”-DN2 population is characterized by the expression of *Mpo* and *Bcl11b* and gives rise in the middle stage of DN2, while in the DN3 population the expression of Flt3, Kit, and Spi1 is absent (Zhou et al., 2019). Subsequently, Naïve T population that double CD4 and CD8 positive cells are generated from the DN4 subpopulation. By expressing T cell receptor, alpha/beta T cells acquire maturation (the formation of CD4^+^ T or CD8^+^ T) in the thymus (Miller, 1961), then act as various types of effector T cells in the peripheral blood system (Fang et al., 2018).

The role of GATA2 in lymphoid lineage differentiation is poorly described. However, GATA2 deficiency patients do present with B/NK lymphopenia and inversions of the CD4/CD8 T cell ratio have been observed (Mutsaers et al., 2013; Ganapathi et al., 2015; Calvo and Hickstein, 2023). So far, there is some evidence that GATA2 plays a role in T cell development in a mouse study showing that loss of the intronic enhancer leads to defects in MPP3 resulting in defective T cell development (You et al., 2022). Interestingly, GATA2 plays a key role in lymphatic vessel and valve formation through binding with the key lymphatic transcriptional regulator Prox1, Foxc2, and Neatc1 in mice (Kazenwadel et al., 2012; Yamazaki et al., 2014; Kazenwadel et al., 2015). But if and how this influences the CD4/CD8 ratio is unclear. B cell lineage differentiation defects were observed in a zebrafish model for GATA2 deficiency syndrome after deletion of Gata2b (Avagyan et al., 2021; Gioacchino et al., 2021b). Gata2b deficiency resulted in increased lymphoid differentiation, but incomplete B cell differentiation due to a loss of B cell lineage transcription factor accessibility. What the underlying molecular mechanism is, is still under investigation.

## 3 The challenges of modelling defects in cell fate determination of GATA2 deficiency syndrome

Human primary cells remain a treasured resource and are widely used to understand the molecular processes underlying hematopoietic cell fate. For instance single cell RNA sequencing from primary patient samples showed clear lineage differentiation defects that reflect the defects observed in patients due to increases in Mk/erythroid priming genes such as *GATA1* and decreases in myeloid priming genes and lymphoid priming genes such as *SPI1* originating in the HSPC population (Wu et al., 2020). Furthermore, recent methylation data from GATA2 deficiency patient samples clearly distinguished symptomatic and asymptomatic patients from healthy donors and highlighted the changes in methylation that underlie leukemia development in these patients (Marin-Bejar et al., 2023).

Unfortunately, due to genetic heterogeneity and the inability to genetically alter these cells, a true comparative study remains impossible with primary cells. Therefore, there will always be a need for model systems to study human disease. Current zebrafish and mouse models of GATA2 deficiency syndrome were only able to partially phenocopy the lineage differentiation defects observed in patients (Dobrzycki et al., 2020; Abdelfattah et al., 2021; Gioacchino et al., 2021a; Avagyan et al., 2021; Gioacchino et al., 2021b; You et al., 2022; Mahony et al., 2023). This could be due to significant disparities between animal models and humans concerning adult size, aging and niche components. The differences may be caused by variations in the quantities of hematopoietic progenitor cells (HPCs) and osteoblasts between these species (Jung et al., 2005; Asada et al., 2015). Differences in population sizes may have impact on the concentration of signaling molecules in human and mouse bone marrow. Furthermore, there are significant differences in cytokine release by the various niche components and differences in cytokine requirements of HSPCs between mouse and human (Scheerlinck, 2014).

To solve these barriers, *ex vivo* human cell models can be used like induced pluripotent stem cells (iPSCs) and embryonic stem cells (ESCs). In iPSCs and ESCs the embryonic hematopoiesis can be simulated, but up to a limited extent. The three waves of embryonic hematopoiesis, from 1) early primitive hematopoiesis, to 2) definitive progenitor hematopoiesis, to 3) definitive HSC formation can only be partially simulated (Sturgeon et al., 2014; Ng et al., 2016; Dzierzak and Bigas, 2018), and the formation of definitive HSCs has not been documented to date. A clear advantage of these models is the fact that these cell models can easily be genetically altered. Atkins et al. also utilized both human ESCs and iPSCs to understand human primitive embryonic hematopoiesis and further detailed the developmental mechanisms of erythroid-myeloid progenitor and lymphoid lineages (Atkins et al., 2022). iPSCs have been used to investigate lineage development (Carcamo-Orive et al., 2017), of B cell (Zhang et al., 2022), T cell (Wang Z. et al., 2022c), NK cell (Zhu et al., 2020; Woan et al., 2021), erythroid lineage (Xin et al., 2021) and myeloid lineage (Mulero-Navarro et al., 2015), providing insights into the molecular mechanisms of various lineages decisions, cellular maturation and cell function. Besides modelling normal hematopoiesis, pluripotent stem cells have been used to study malignant hematopoiesis. Patient-specific iPSCs have been used to understand the mechanisms of leukemic transformation, as well as screening patient-specific drugs (Turhan et al., 2019; Olofsen et al., 2020; Bigas et al., 2022; Olofsen et al., 2023). It must be noted that iPSC of patients with GATA2 mutations showed only marginal differences. Specifically, GATA2 patient-specific iPSCs exhibited nuanced differentiation phenotypes dependent upon the tissue which the iPSCs were derived from. Hematopoietic maturation was reduced from iPSC where GATA2 was mutated using CRISPR/Cas9. This heterogeneity in differentiation outcomes hampers the investigation of the role of GATA2 in lineage differentiation using this model system (Jung et al., 2018).

In addition to employing human cell models, the utilization of humanized animal models represent a valuable method to investigate the functional role of GATA2 in hematopoietic lineage determination. The most common humanized animal model to study hematopoiesis is the NSG immunodeficient mouse model, which allows us to study the mechanism of hematopoietic lineage determination with human cells *in vivo* (Adigbli et al., 2020). By xenotransplantation, it is possible to trace the differentiation of HSPCs carrying GATA2 mutations. However, as previously elucidated, it is crucial to consider the impact of microenvironmental components on hematopoiesis. Although the differentiation of human HSPCs can be activated in mouse bone marrow by the expression of human cytokines, the biological difference between mouse and human should be considered and may not represent the best model to study lineage differentiation defects.

Another complication in the study of GATA2 deficiency syndrome is the vast variety between patients. Some patients remain asymptomatic, while others suffer from immune deficiencies and yet others develop myeloid malignancies at an early age. Important considerations are the many different mutation types that are found between these patients, but also the environmental factors that contribute to our health, i.e., secondary injuries like infection. Inflammation has been recognized as driver of leukemogenesis and could contribute to disease progression and the variety observed between these patients (Essers et al., 2009; Rodriguez-Meira et al., 2023). A more likely model for GATA2 deficiency may thus be the current mouse models with an addition of a secondary injury like transplantation or stimulation with LPS, know to induce inflammation (Abdelfattah et al., 2021).

## 4 Conclusion and discussion

GATA2 has a pivotal role in HSC self-renewal and hematopoietic lineage determination. The precise expression regulation of GATA2 has a profound impact on hematopoietic development, as high expression of GATA2 is required for HSC self-renewal and maintenance, while procedural downregulation is imperative to facilitate downstream lineage differentiation. Recent advances in the field in terms of new animal models to understand the precise role of GATA2 in lineage differentiation can significantly contribute to the development of treatments for the life-threatening cytopenias from which the majority of GATA2 deficiency syndrome patients suffer (Gioacchino, et al., 2021a; Gioacchino et al., 2021b; Mahony et al., 2023). In recent years, the employment of single cell sequencing technologies resulted in remarkable progress in the comprehension of the molecular regulatory processes governing hematopoietic cell fate determination (Ranzoni et al., 2021; Zeller et al., 2023; Zhang et al., 2023). These technological advancements have supported and continue to support the deconstruction of the functions of GATA2 in hematopoiesis, and the pathophysiological mechanisms behind GATA2 deficiency syndrome. The precise mechanism behind GATA2 deficiency-related immunodeficiency, the variation between patients and the progression to myeloid leukemia remains to be elucidated. Furthermore, considering the different genetic backgrounds and inflammatory burden, caution should be exercised when addressing research questions and conclusions between human and animal models. Thus, there is a need to continue the development of animal or human cell-based research models.
